# Macular Pigment and Open-Angle Glaucoma in the Elderly: The Montrachet Population-Based Study

**DOI:** 10.3390/jcm11071830

**Published:** 2022-03-25

**Authors:** Louis Arnould, Alassane Seydou, Christine Binquet, Pierre-Henry Gabrielle, Chloé Chamard, Lionel Bretillon, Alain M. Bron, Niyazi Acar, Catherine Creuzot-Garcher

**Affiliations:** 1 Department of Ophthalmology, Dijon University Hospital, 21000 Dijon, France; phgabrielle@gmail.com (P.-H.G.); alain.bron@chu-dijon.fr (A.M.B.); catherine.creuzot-garcher@chu-dijon.fr (C.C.-G.); 2 Clinical Epidemiology/Clinical Trials Unit, Clinical Investigation Center, INSERM, CIC 1432, Dijon University Hospital, 21000 Dijon, France; christine.binquet@u-bourgogne.fr; 3 Centre des Sciences du Goût et de l’Alimentation, AgroSup Dijon, CNRS, INRAE, Université Bourgogne Franche-Comté, 21000 Dijon, France; alassanemaiga7@yahoo.fr (A.S.); lionel.bretillon@inrae.fr (L.B.); niyazi.acar@inrae.fr (N.A.); 4 Department of Ophthalmology, Gui de Chauliac Hospital, 34000 Montpellier, France; chloe.chamard@gmail.com; 5 Neuropsychiatry—Epidemiological and Clinical Research, Inserm, PSNREC, Montpellier University, 34000 Montpellier, France

**Keywords:** mcular pigment optical density, glaucoma, two-wavelength autofluorescence, elderly, population-based study

## Abstract

(1) Background: To compare macular pigment optical density (MPOD) and its spatial distribution between eyes with primary open-angle glaucoma (POAG) and control eyes in an elderly population. (2) Methods: The Montrachet study (Maculopathy Optic Nerve and nutrition neurovAsCular and HEarT) is a population-based study including participants aged 75 years and over. All participants had a slit lamp examination, fundus photographs, and a questionnaire about their medical past history and smoking status. Optic disc spectral domain optical coherence tomography was also performed. All glaucoma-suspected patients were convocated to have a new full examination. We only retained one eye with POAG for analysis in the glaucoma group and one eye without optic neuropathy in the control participants group. MPOD measurements were performed with the two-wavelength autofluorescence method (488 and 514 nm). (3) Results: Overall, 601 eyes had MPOD measurements among 1153 participants. Among the 601 eyes, 48 had POAG. The mean age for the glaucoma and control participants was 84.01 ± 4.22 years and 81.94 ± 3.61 years, respectively (*p* < 0.001). In the multivariable analysis, we could not find any association between POAG and MPOD at 0.5° (*p* = 0.336). We found no significant difference regarding MP spatial distribution between the two groups (*p* = 0.408). (4) Conclusion: In this elderly population-based study, eyes with POAG and control eyes without optic neuropathy did not differ in terms of MPOD and MP spatial distribution.

## 1. Introduction

Macular pigment (MP) plays an important role in visual function [1] and in the protection of the retina against oxidative damage [2,3]. It is located in the inner layers of the retina [4] and composed of three carotenoids. Two of them are exclusively from dietary origin (lutein and zeaxanthin) while the third one (meso-zeaxanthin) is synthetized from lutein. The highest MP concentration is located in the Henle’s fiber layer of the fovea and rapidly decreases to be undetectable outside the macula. In the literature, three MP patterns have been reported: the no-ring profile, ring-like profile, and an intermediate profile [5,6,7,8]. MP and oral dietary carotenoid supplementation have been extensively studied in macular disorders, especially in age-related macular degeneration (AMD). Numerous studies suggested the role of carotenoid in the prevention of AMD [9,10].

Glaucoma is the most common optic neuropathy in adulthood, leading to irreversible damages of the structure and function of the optic nerve [11]. It is defined as a multifactorial progressive optic neuropathy with a typically acquired loss of optic nerve fibers (death of the retinal ganglion cell axons) [12,13]. Primary open-angle glaucoma (POAG) is the most common form of this disease [14]. In 2020, it was estimated that nearly 52 million people are affected by POAG worldwide and that this number would increase to 79 million by 2040 [15].

To date, the relationship between MPOD and POAG is conflicting and the association between neurodegenerative disease and MP is less straightforward. MP and glaucoma could be linked based on two major hypotheses: microvascular and oxidative stress processes [16]. According to several research teams, MP could absorb vision blue light, hence decreasing light energy in the inner retina and photo-oxidative injury [17]. Some studies found that lower densities of MP were found in glaucoma patients, [18] whereas other studies could not support this assumption [19]. The primary objective of this study was to compare MPOD between eyes with POAG and control eyes in an elderly population. The secondary objective was to compare the MP spatial distribution between both groups.

## 2. Materials and Methods

### 2.1. Population Study

The Montrachet study (Maculopathy Optic Nerve and nutrition neurovAsCular and HEarT) was an ancillary study of the population-based Three Cities (3C) study, which had been previously reported [20]. In the 3C cohort study, 9294 persons were randomly selected from the electoral rolls of three French urban cities (Bordeaux, Dijon, and Montpellier) and aged 65 years. Ten years later, the subgroup of participants from Dijon was invited to participate in the Montrachet study.

The methodology of the Montrachet study and baseline characteristics of volunteers have already been described [21]. From October 2009 to March 2013, 1153 volunteers underwent complete eye examination in the Department of Ophthalmology of the Dijon University Hospital, France. All participants were asked to complete a questionnaire about their lifestyle (alcohol consumption and smoking status) and environment (sun exposure). This examination included the collection of self-reported eye disease and treatment history, visual acuity measurement, intraocular pressure measurement (Tonoref II, Nidek, Gamagori, Japan), central corneal thickness measurement with an ultrasonic contact pachymeter (DGH 500, DGH Technology, Exton, PA, USA), visual field examination with a screening program (Frequency-Doubling Technology, Carl Zeiss Meditec, Dublin, CA, USA), and spectral-domain optical coherence tomography (SD-OCT; software version 5.4.7.0; Spectralis, Heidelberg Engineering Co., Heidelberg, Germany) for both the macula and the optic nerve head after pupil dilation. The high-speed resolution mode and the eye-tracking system were activated to acquire the images. For the optic disc, the retinal nerve fiber layer (RNFL) thickness acquisition was obtained with a circle diameter of 3.5 mm. Moreover, each participant benefited from two retinal photographs: one centered on the macula and one on the optic nerve (TRC NW6S, Topcon, Tokyo, Japan). In addition, fasting blood samples were drawn to measure plasma carotenoids and fatty acids. We also used a semiquantitative food frequency questionnaire to quantify the dietary intake of lutein and zeaxanthin among participants. The study was approved by the regional ethics committee and was registered as 2009-A00448-49. All participants gave their informed consent. This study adhered to the tenets of the Declaration of Helsinki and we followed the STROBE policy according to the EQUATOR guidelines [22].

### 2.2. Glaucoma Diagnosis

Glaucoma diagnosis in the Montrachet study has already been reported [23,24]. Briefly, optic disc photographs were reviewed by two trained ophthalmologists (LA and PHG) masked for clinical and RNFL thickness information. In case of discrepancy, a senior glaucoma specialist (AMB) made an adjudication. Suspected-glaucoma eyes benefited from a new examination with gonioscopy and a Humphrey Swedish Interactive Thresholding Algorithm 24-2 visual field (Carl Zeiss Meditec, Dublin, CA, USA). Glaucoma was defined by the glaucoma classification of the International Society of Geographical and Epidemiological Ophthalmology (ISGEO) [25]. In case of bilateral disease, the eye with the most severe presentation was kept for analysis. Only cases of primary open-angle glaucoma (POAG) were considered for the present analysis. Secondary glaucoma, angle-closure glaucoma, and non-glaucomatous optic neuropathy were excluded from the analysis.

### 2.3. MPOD Measurements

The MPOD was measured with the two-wavelength autofluorescence method using a modified Heidelberg Retina Angiograph (HRA; Heidelberg Engineering, Heidelberg, Germany). After pupil dilation with tropicamide 0.5% (Thea, Clermont-Ferrand, France), two acquisitions were performed at a 30 s interval and captured at 488 and 514 nm excitation wavelengths by a trained technician for both eyes (Figure S1). Glaucomatous status was masked to the operator. MPOD maps were generated by digital subtraction of the log autofluorescence images. We recorded MPOD at 0°, 0.5°, 1°, 2°, and 6° eccentricities using the software provided by the manufacturer of the device. MPOD was expressed in optical density units (DU). In the control participants group without optic neuropathy, the eye with the best image quality was retained for analysis. The right eye was chosen when image quality was similar in both eyes. We excluded participants with poor image quality in both eyes and those who suffered from late age-related macular degeneration (AMD). Classification of late AMD was based on both color fundus and OCT images [26]. From the graphs generated by the software of the modified HRA, we divided the different MP spatial distribution profiles into three groups (ring-like, no ring, and intermediate). A second investigator (PHG) analyzed fifty eyes of our population randomly chosen independently from the first investigator (LA).

### 2.4. Blood Sampling

Blood samples were collected from fasted volunteers for plasma lipids and fatty acids analysis [27]. Lipids extracted from plasma were stored under inert gas and then transmethylated with boron trifloride in methanol [28]. Finally, fatty acid methyl esters were isolated with hexane and analyzed by gas chromatography using the Hewlett Packard Model 5890 (Palo Alto, CA, USA) with a CPSIL-88 column (100 m × 0.25 mm i.d., fim thickness 0.20 µm; Varian, Les Ulis, France) equipped with a flame ionization detector. The carrier gas used was hydrogen.

### 2.5. Statistical Analysis

Categorical variables were expressed as a number (*n*, percentage) and continuous variables as mean ± standard deviation (SD) or median (interquartile range (Q1–Q3)) according to their distribution. We performed our analysis with one eye per individual as the unit of analysis. We displayed two groups: control eyes and eye with POAG. Bivariate analysis was performed with Pearson’s chi-square or Fisher exact tests for percentage comparisons as appropriate. Student test or analyses of variance, or the Kruskal–Wallis test was used for comparison of mean or median when appropriate. Cohen’s kappa coefficient was used to measure the concordance between the two eyes for MPOD and for MP spatial distribution profiles between the two investigators. For multivariable analysis between MPOD at 0.5° and the presence of POAG as a dependent variable, all variables associated with glaucoma in the bivariate analysis with a *p*-value < 0.20 were included in the model. The smoking status variable was forced in the model. Then, a final model adjusted for age, sex, smoking status, lens status, plasma alpha-linoleic acid (ALA), and eicosapentaenoic acid (EPA) n-3 polyunsaturated fatty acids (PUFAs) was obtained by manual backward selection. For all analyses, the tests were two-tailed and the results were considered significant when *p*-values were less than 0.05. Analyses were performed using SAS software (version 9.4; SAS Institute, Inc., Cary, NC, USA).

## 3. Results

### 3.1. Demographics and Baseline Characteristics

**Figure 1:**
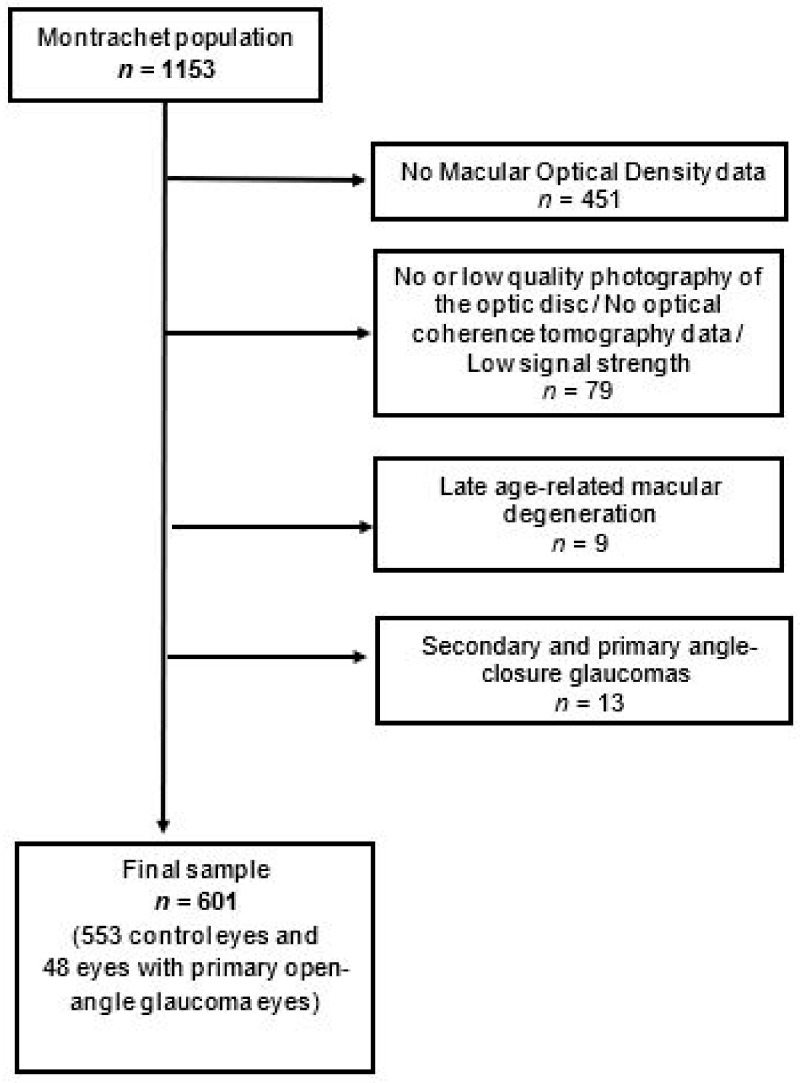
Eyes (553 eyes without optic neuropathy and 48 eyes with POAG). The baseline demographics and clinical characteristics of participants and non-participants are shown in Table 1.

A total of 702 participants had MPOD measurements among the 1153 from the Montrachet study (Figure 1).

Non-participants were more likely to be phakic and to present late AMD. Participants’ mean age was 82.11 ± 3.70 years.

The characteristics of the study population are displayed in Table 2.

The POAG patients were more likely to be men and they were significantly older. The eyes with POAG were more likely to be pseudophakic, to present with a larger cup-to-disc ratio and a thinner global RNFL thickness, and with a negative spherical equivalent.

### 3.2. MPOD and Primary Open-Angle Glaucoma

The concordance between the two eyes for MPOD among 50 randomly chosen subjects found an agreement with kappa 0.79 (confidence interval = 0.64–0.95). The MPOD means at the 0°, 0.5°, 1°, 2°, and 6° eccentricities for the two groups are presented in Table 3.

In this univariate analysis, there was no statistically significant difference at each eccentricity degree between the two groups. After adjustment for age, gender, smoking and lens status, and plasma PUFAs, we compared the MPOD at 0.5° eccentricity between eyes without optic neuropathy and eyes with POAG. We could not find a statistically significant difference (*p* = 0.336; Table 4).

Similarly, we did not find a statistical difference for MPOD at 1°, 2°, and 6° eccentricity (data not shown). In the multivariable analysis, older age (*p* = 0.041), male sex (*p* = 0.017), pseudophakic status (*p* = 0.044), and the elevated plasma level of ALA (*p* = 0.017) were significantly associated with the presence of POAG. The agreement for MP spatial distribution profiles between the two investigators was 0.71 (confidence interval = 0.48–0.93). MP spatial distribution was classified into three patterns, namely ring-like (10.64%), intermediate (8.51%), and no ring-like (80.85%) in eyes with POAG as well as 17.90%, 10.67%, and 71.43% in the control eyes. There was no significant difference regarding the MP spatial distribution between eyes with POAG and eyes without optic neuropathy (*p* = 0.408; Table 2).

## 4. Discussion

Our study was designed to compare MPOD in an elderly population between POAG and control eyes using the two-wavelength autofluorescence method. Additionally, we aimed to investigate the MP spatial distribution between these two groups. We found no difference of MPOD and MP spatial distribution between eyes with POAG and eyes without optic neuropathy. These results are not in agreement with previous studies [16,29,30]. Igras E. et al. found a statistically significant difference for MPOD between glaucoma patients and controls with a MPOD median of 0.23 (−0.19; 0.65) and 0.36 (−0.08; 0.80) at 0.5° eccentricity, respectively [16]. Ji Y. et al. found MPOD significantly reduced in the glaucoma group compared to the control group, with 0.116 ± 0.033 UD and 0.137 ± 0.026 UD, respectively [30]. Recently, Siah WF. et al. [29] found that MPOD was lower in glaucomatous eyes with foveal ganglion cell complex involvement. We should note that these three case-control studies focused on smaller samples of selected patients (*n* = 40, *n* = 30, and *n* = 88, respectively). Nevertheless, our results could be difficult to compare to other studies as the MP measurement procedures were different. We measured MP with a two-wavelength autofluorescence method whereas Siah WF. et al. [29], Igras E. et al. [16], and Ji Y. et al. [30] used customized heterochromatic flicker photometry (cHFP) and a fundus reflectance method. On one hand, the reflectance method has some limitations because it uses only one wavelength, which is problematic in the case of lens opacification [31]. On the other hand, cHFP results should be interpreted with caution as it is influenced by the operator’s execution and participants’ cooperation [32]. Moreover, cHFP is a time-consuming technique (approximately 30 min), which is difficult to realize in elderly participants. The two-wavelength autofluorescence method for MPOD measurement is faster (2 to 3 min for each participant) and objective, and can be performed by trained technicians. Nevertheless, MP values obtained by means of the Heidelberg Spectralis (HRA) with the two-wavelength autofluorescence method were comparable to MP values obtained using the densitometer (cHFP) [33].

In contrast to the three previous research groups, our results are in line with Daga FB and Bruns Y et al. [19,34]. Daga FB et al. found that patients diagnosed with glaucoma (mean age of 72.5 years) had comparable MP levels to control subjects (mean age of 70.0 years) with the same two-wavelength autofluorescence method. Moreover, they also demonstrated no significant relationship between MP, standard automated perimetry, and retinal nerve fiber layer thickness measurements [19]. Bruns Y et al. presented that there was no evidence for lower MPOD in their 43 glaucomatous-patient (mean age of 70.0 years) case-control study [34]. These conflicting results could also be found in epidemiology studies. Jae H. Kang et al. presented in a large population-based study (Nurses’ Health Study and Health Professionals Follow-up Study) that increased dietary levels of carotenoid were associated with a lower risk of POAG [35]. On the other hand, in the Rotterdam study, no protective effect was found between carotenoids and POAG [36].

A negative correlation has been demonstrated between BMI and MPOD but in our study, we did not show any significant difference between the controls and POAG participants regarding their BMI [37].

In our study, the eyes with POAG were more likely to be pseudophakic, which could have influenced the MPOD measurement as mentioned by Sasamoto et al. [38]. Nevertheless, the difference between the phakic and pseudophakic eyes found in the literature is mainly due to the absorption of blue light by the cataractous lens. Thus, we took into account the lens status in the multivariable analysis in order to limit misinterpretation of the MPOD measurement secondary to blurred media due to cataract.

Carotenoids supplementation is a confounding factor for MPOD because it has been reported that supplementation increased the concentration of carotenoids in plasmatic serum and MPOD [39,40]. Unfortunately, we were not able to evaluate this point as there was no lutein/zeaxanthin supplementation in the POAG group. We used a semiquantitative food frequency questionnaire to quantify the dietary intake in terms of carotenoid among participants in order to control for any disparity in MPOD caused by diet. In our study, there was no significant difference between the two groups in term of carotenoids intake (data not shown).

One school of thought suggests from preclinical studies of the glaucoma model that MP could operate as a neuroprotective agent. MP could regulate the production of pro-oxidant stressors in the early ischemic retinal injury and protect the inner retina from the neurodegenerative process [41,42]. Another school of thought strengthened by the results of the present study found no association between MP and glaucoma [19,34,36]. Hence, positive results could be explained by many confounders (healthy diet and lifetime exposure) and not independently by carotenoids and MP.

We found a significant difference between glaucomatous and control participants for age, gender, and lens status. These results are in agreement with previous studies which showed that POAG prevalence increased with age and men were more likely to present with POAG compared to women [43,44,45]. Regarding lens status and POAG prevalence, results in the literature are not clear as in some situations, cataract surgery is part of the treatment of POAG. Significative association between an elevated plasma level of ALA and POAG is conflicting. It was previously presented that there was no significant difference regarding isolated plasma FAs between participants with POAG and participants without optic neuropathy in elderly [46]. Considering the dispersion of the results for elevated plasma levels of ALA and POAG in this study, this association should be confirmed by further clinical studies.

We acknowledge several limitations of this study. First, we analyzed only 611 eyes of 1153 participants in the Montrachet study due to imaging quality and availability of MP evaluation. Thus, missing data could decrease the power of our analysis even though there was no difference between participants and non-participants. Second, these findings are based on a Caucasian European population and cannot be extrapolated to other parts of the world and other ethnicities. Third, this exploratory cross-sectional study only enhanced a potential absence of association between MPOD, MP spatial distribution, and glaucomatous optic neuropathy. This should be confirmed by a longitudinal study to validate our findings. Fourth, it is also important to acknowledge that our two groups are unequal (*n* = 48 versus *n* = 553) with a small sample size of glaucomatous patients, which could lead to statistical bias and lower statistical power. Fifth, we did not collect ganglion cell layer thickness measurements, which could have given us the opportunity to focus the analysis on glaucomatous eyes with foveal ganglion cell complex loss.

We thought that the strengths of a population-based study could help to clarify the debate on the relationship of MP and glaucoma. In contrast to control-case studies, participants were enrolled in the Montrachet study regardless of their glaucomatous status.

## 5. Conclusions

In conclusion, our study investigated the difference of MPOD and MP spatial distribution between POAG eyes and control eyes in the elderly in the frame of a population-based study. We found no significant difference between these two groups. The clinical relevance of the relationship between POAG and macular pigment warrants further studies.

## Figures and Tables

**Table 1:** Baseline characteristics between participants and non-participants in the Montrachet study.

	Total (*n* = 1153)	Participants	Non-Participants	
		(*n* = 601)	(*n* = 552)	*p*-Value
Age, years	82.25 ± 3.75	82.11 ± 3.70	82.41 ± 3.76	0.165
Gender				
Men	430 (37.29)	232 (38.60)	190 (35.87)	0.339
Smoking status	390 (34.42)	208 (35.25)	182 (33.52)	0.539
Alcohol consumption	64 (6.29)	31 (5.87)	33 (6.75)	0.565
Body mass index, kg/m^2^				
<25	598 (51.86)	315 (52.41)	283 (51.27)	0.698
≥25	555 (48.14)	286 (47.59)	269 (48.73)	
Central retinal thickness, µm	292.51 ± 49.56	293.60 ± 44.55	293.40 ± 54.59	0.942
Cup-to-disc ratio	0.34 ± 0.21	0.34 ± 0.22	0.33 ± 0.21	0.407
Spherical equivalent, diopters	0.06 ± 2.11	0.13 ± 2.06	−0.03 ± 2.18	0.241
Lens status				
Phakic	583 (50.70)	274 (45.59)	309 (56.28)	<0.001
Pseudophakic	567 (49.30)	327 (54.41)	240 (43.72)	
Sun protection				
Never	115 (10.02)	60 (10.02)	55 (10.02)	0.799
Occasionally	258 (22.47)	130 (21.70)	128 (23.32)	
Often	775 (67.51)	409 (68.28)	366 (66.67)	
Iris color				
Blue/gray	465 (40.33)	244 (40. 60)	221 (40.04)	
Green/hazel	360 (31.22)	202 (33.61)	158 (28.62)	0.067
Dark brown/black	328 (28.45)	155 (25.79)	173 (31.34)	
AMD stages				
No AMD	587 (54.96)	322 (54.95)	265 (54.98)	<0.0001
Early AMD stage 1	337 (31.55)	198 (33.79)	139 (28.84)	
Early AMD stage 2	100 (9.36)	53 (9.04)	47 (9.75)	
Early AMD stage 3	22 (2.06)	13 (2.22)	9 (1.87)	
Late AMD	22 (2.06)	0 (0.00)	22 (4.56)	
Plasma xanthophylls, µg/L				
L	271.41 (178.07–453.91)	277.87 (177.89–448.29)	266.59 (179.10–454.84)	0.863
Z	17.81 (11.28–26.00)	17.39 (11.49–26.32)	18.17 (10.65–25.63)	0.946
L/Z supplementation, yes	65 (5.64)	34 (5.56)	31 (5.72)	0.909
Plasma n-3 PUFAs				
ALA	0.63 ± 0.21	0.63 ± 0.21	0.63 ± 0.22	0.859
EPA	1.29 ± 0.62	1.32 ± 0.65	1.25 ± 0.58	0.084
DHA	2.22 ± 0.65	2.27 ± 0.67	2.16 ± 0.62	0.022
Plasma lipids, mmol/L				
Total cholesterol	5.83 ± 0.93	5.83 ± 0.93	5.77 ± 0.97	0.324
LDL cholesterol	3.60 ± 0.83	3.62 ± 0.83	3.58 ± 0.84	0.511
HDL cholesterol	1.66 ± 0.40	1.66 ± 0.40	1.67 ± 0.41	0.869
Triglycerides	1.17 ± 0.52	1.20 ± 0.54	1.14 ± 0.49	0.055

*p*-value was calculated between participants and non-participants. The results are displayed as *n* (%) for categorical variables and as mean ± standard deviation or as median (interquartile range (IQR)) depending on their distribution for continuous variables. AMD = age-related macular degeneration; L = lutein; Z = zeaxanthin; PUFA = polyunsaturated fatty acids; ALA = alpha-linoleic acid; EPA = eicosapentaenoic acid; DHA = docosahexaenoic acid; LDL = low-density lipoprotein; and HDL = high-density lipoprotein. Missing data for smoking status (current or past, *n* = 20), alcohol consumption (current or past *n* = 136), central retinal thickness (*n* = 8), cup-to-disc ratio (*n* = 105), spherical equivalent (*n* = 103), lens status (*n* = 3), sun protection (*n* = 5), AMD stages (*n* = 85), L/Z (*n* = 358), plasma n-3 PUFAs (*n* = 339), LDL cholesterol (*n* = 14), HDL cholesterol (*n* = 14), and triglycerides (*n* = 15).

**Table 2:** Baseline characteristics of eyes without optic neuropathy and eyes with primary open-angle glaucoma in the Montrachet study, *n* = 601.

	Eyes without Optic Neuropathy (*n* = 553)	Eyes with POAG (*n* = 48)	*p*-Value
Age, years	81.94 ± 3.61	84.01 ± 4.22	<0.001
Gender			
Men	206 (37.25)	26 (54.17)	0.021
Smoking status	191 (35.11)	17 (36.96)	0.801
Alcohol consumption	29 (5.97)	2 (4.76)	0.749
BMI, kg/m^2^			
<25	291 (52.62)	24 (50.00)	0.727
≥25	262 (47.38)	24 (50.00)	
Central retinal thickness, µm	292.90 ± 43.29	302.30 ± 56.92	0.158
Global RNFL thickness, μm	95.3±11.7	79.6±19.0	<0.001
Cup-to-disc ratio	0.31 ± 0.19	0.73 ± 0.10	<0.001
Intraocular pressure, mmHg	15.49 ± 3.32	15.46 ± 3.32	0.407
Mean deviation of visual field, dB	NA	−11.69 ± 8.12	NA
Spherical equivalent, diopters	0.20 ± 2.04	−0.76 ± 2.09	0.002
Best-corrected visual acuity, <20/60	12 (2.2)	2 (4.2)	0.428
Lens status			
Phakic	264 (47.74)	10 (20.83)	<0.001
Pseudophakic	289 (52.26)	38 (79.17)	
Sun protection			
Never	58 (10.51)	2 (4.26)	0.357
Occasionally	118 (21.38)	12 (25.53)	
Often	376 (68.11)	33 (70.21)	
Macular pigment distribution			
No ring	395 (71.43)	38 (80.85)	0.408
Intermediate	59 (10.67)	4 (8.51)	
Ring-like	99 (17.90)	5 (10.64)	
Iris color			
Blue/gray	224 (40.51)	20 (41.67)	0.158
Green/hazel	191 (34.54)	11 (22.92)	
Dark brown/black	138 (24.95)	17 (35.41)	
AMD stages			
No AMD	296 (54.82)	26 (56.53)	0.625
Early AMD stage 1	184 (34.07)	14 (30.43)	
Early AMD stage 2	47 (8.70)	6 (13.04)	
Early AMD stage 3	13 (2.41)	0 (0.00)	
Plasma xanthophylls, µg/L			
L	269.58 (176.846–443.43)	316.38 (180.00–435.20)	0.625
Z	17.44 (11.49–26.32)	15.78 (11.49–27.48)	0.905
L/Z supplementation, yes	31 (5.61)	0 (0.00)	NA
Plasma n-3 PUFAs			
ALA	0.63 ± 0.21	0.70 ± 0.26	0.057
EPA	1.34 ± 0.66	1.14 ± 0.35	0.068
DHA	2.27 ± 0.69	2.22 ± 0.45	0.628
Plasma lipids, mmol/L			
Total cholesterol	5.84 ± 0.94	5.69 ± 0. 80	0.311
LDL cholesterol	3.62 ± 0.84	3.59 ± 0.77	0.836
HDL cholesterol	1.67 ± 0.39	1.58 ± 0.45	0.134
Triglycerides	1.21 ± 0.55	1.15 ± 0.40	0.468

*p*-value was calculated between participants and non-participants. The results are displayed as *n* (%) for categorical variables and as mean ± standard deviation or as median (interquartile range (IQR)) depending on their distribution for continuous variables. POAG = primary open-angle glaucoma; RNFL = retinal nerve fiber layer; AMD = age-related macular degeneration; L = lutein; Z = zeaxanthin; NA = not applicable; PUFA = polyunsaturated fatty acids; ALA = alpha-linoleic acid; EPA = eicosapentaenoic acid; DHA = docosahexaenoic acid; LDL = low-density lipoprotein; and HDL = high-density lipoprotein. Missing data for smoking status (*n* = 11), alcohol consumption (*n* = 73), sun protection (*n* = 2), central retinal thickness (*n* = 1), cup-to-disc ratio (*n* = 7), macular pigment distribution (*n* = 1), AMD stages (*n* = 15), plasma L/Z (*n* = 164), plasma n-3 PUFAs (*n* = 154), LDL cholesterol (*n* = 6), HDL cholesterol (*n* = 6), and triglycerides (*n* = 6).

**Table 3:** Macular pigment optic density at several degree eccentricities from the fovea in control and primary open-angle glaucoma eyes in the Montrachet study.

	Total (*n* = 601)	Eyes without Optic Neuropathy (*n* = 553)	Eyes with POAG (*n* = 48)	*p*-Value
MPOD at 0°	0.73 ± 0.32	0.72 ± 0.32	0.77 ± 0.30	0.308
MPOD at 0.5°	0.56 ± 0.26	0.55 ± 0.26	0.61 ± 0.25	0.165
MPOD at 1°	0.48 ± 0.22	0.47 ± 0.22	0.51 ± 0.21	0.345
MPOD at 2°	0.30 ± 0.14	0.30 ± 0.14	0.32 ± 0.13	0.427
MPOD at 6°	0.07 ± 0.04	0.07 ± 0.04	0.08 ± 0.04	0.505

*p*-value was calculated between control and primary open-angle glaucoma eyes. The results are displayed as mean ± standard deviation. POAG = primary open-angle glaucoma and MPOD = macular pigment optical density. MPOD was measured in optical density units.

**Table 4:** Multivariable analysis of association between MPOD at 0.5° and the presence of primary open-angle glaucoma in the Montrachet study.

	OR (95% CI)	*p*-Value
Age, years	1.10 (1.01–1.20)	0.041
Sex, male	2.73 (1.19–6.25)	0.017
Smoking status, yes	0.60 (0.25–1.43)	0.251
Lens status, pseudophakic	2.49 (1.01–6.16)	0.044
Plasma PUFAs		
ALA	7.16 (1.42–36.16)	0.017
EPA	0.47 (0.21–1.04)	0.063
MPOD at 0.5°	2.08 (0.47–9.23)	0.336

PUFA = polyunsaturated fatty acids; ALA = alpha-linoleic acid; EPA = eicosapentaenoic acid; MPOD = macular pigment optical density; OR = odds ratio; and CI = Confidence interval. In total, 162 observations were deleted due to missing values from smoking status, lens status, and plasma PUFAs. MPOD was measured in optical density units.

## Data Availability

Data are available upon reasonable request. A Three Cities data request form has to be sent to louis.arnould@chu-dijon.fr.

## Supplementary Materials

The following supporting information can be downloaded at: https://www.mdpi.com/article/10.3390/jcm11071830/s1, Figure S1: A digital subtraction image with intensity corresponding to MPOD (right) and their measurements plotted from center to periphery of fovea corresponding to the mean MPOD at each eccentricity (left).
