# Supplementary material for: Macular Pigment and Open-Angle Glaucoma in the Elderly: The Montrachet Population-Based Study
Source: J Clin Med. 2022 Mar 25;11(7):1830. doi: 10.3390/jcm11071830 (PMC8999647; doi:10.3390/jcm11071830)
Supplement: Supplementary file 1 [file jcm-11-01830-s001.zip › jcm-1648533-supplementary.pdf]

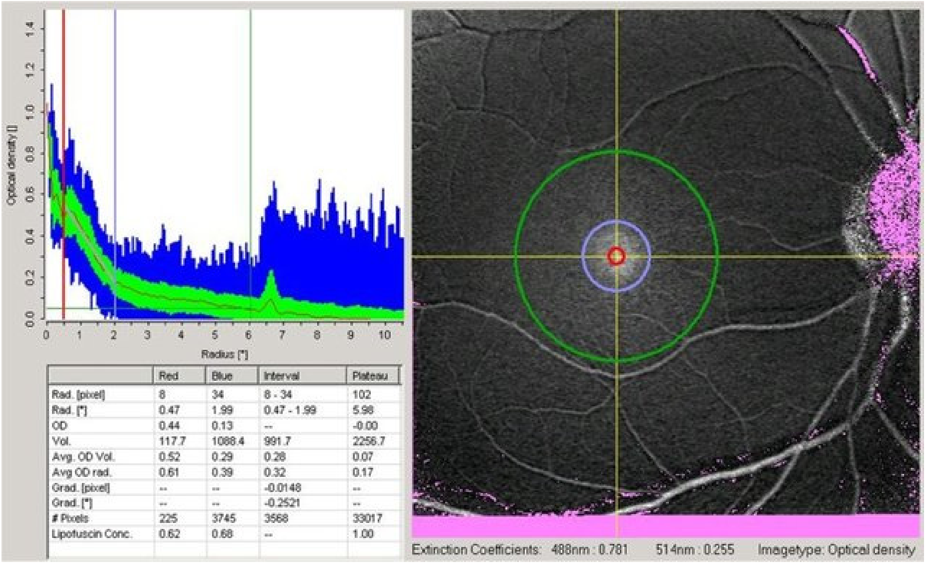

Figure S1. A digital subtraction image with intensity corresponding to MPOD (right) and their measurements plotted from center to periphery of fovea corresponding to the mean MPOD at each eccentricity (left).
